# The Bayes Factor, a Suitable Complement beyond Values of *p*<0.05 in Nursing Research and Education

**DOI:** 10.17533/udea.iee.v39n1e14

**Published:** 2021-03-05

**Authors:** Cristian Antony Ramos-Vera

**Affiliations:** 1 Bachelor's degree in psychology. Research Area, Faculty of Health Sciences. Universidad Cesar Vallejo, Lima (Perú). Email: cristony_777@hotmail.com Universidad César Vallejo Faculty of Health Sciences Universidad Cesar Vallejo Lima Peru cristony_777@hotmail.com

Dear Madame Editor:

In accordance with a recent study in the current journal, significant differences are reported on burnout related with COVID-19 in two groups of nurses with and without experience with patients with COVID-19 infection in Iran, by using Student’s statistical t test,(1) It reports that experienced frontline nurses exposed to treating COVID-19 patients indicate higher levels of job stress and burnout. This comparative analysis is among the most used in medical sciences based on the null hypothesis significance test (NHST) according to the “*p* <0.05” significance level that infers rejection of the null hypothesis (no difference) and provides greater likelihood confidence to researchers to assume the alternate hypothesis (difference) given the study sample.(2)

Bayesian statistics also permits contrasting hypotheses through probabilities of credibility, being a suitable complement to reinforce statistical significance and, when having frequentist significant findings, it is also a methodological alternative of statistical replication.(3-5) From the Bayesian model, the Bayes factor is the inclusive method of *a priori* and *posteriori* credibility to evaluate beyond the level of significance, given that it estimates the degree to which the data support the statistical hypotheses, from Jeffreys’ classification scheme for Student’s t analysis(5^,^^6^) “weak”, “moderate”, “strong”, and “very strong” (Table 1).

When having prior significant results, the only requirement is the t value (-2.86), as well as the sample sizes of both groups (151 and 94) reported by Hoseinabadi *et al.*(1) Regarding the Bayes factor, it permits inferring two interpretations: FB_10_ (in favor of the alternative hypothesis of significant difference) and BF_01_ (in favor of the null hypothesis from lack of significant difference) and the 95% confidence interval.(4) Upon the evidence of significant difference, this analysis focuses on estimating the degree of certainty of the alternate hypothesis. The results obtained through the Bayes factor are BF_10_ = 6.529 and BF_01_ = 0.153, with 95%CI [-0.388 to -0.068]. These findings report moderate evidence in favor of the statistical hypothesis of significant difference; this may be interpreted in that the alternate hypothesis is six times greater than the nullity of the data and it - in turn - is reduced proportionally for some possible interpretation. Likewise, the parameter is reported of the maximum Bayes factor (maxBF_10_ = 8.444) to determine the stability of the results, which indicates a greater support magnitude to the statistical differences, endorsing the reliability of the findings obtained.(4^,^^7^)

**Table 1:** Values of quantifiable interpretation of the Bayes factor*

Value	Interpretation	Evidence for the hypothesis
>30	Very strong	Alternative
10-30	Strong	Alternative
3.1-10	Moderate	Alternative
1.1-3	Weak	Alternative
1	0	No evidence available
0.3-0.9	Weak	Null
0.3-0.1	Moderate	Null
0.1-0.03	Strong	Null
	Very strong	Null

*Elaborated by the author

Likewise, this Bayesian approach is quite useful in other statistical analyses and re-analyses based on the significance value “*p* <0.05” (correlation, linear regression, logistic regression, ANOVA), which only has a pair of recent guides that permit disseminating the use and interpretation of the Bayes factor beyond nursing research, encompassing its relevance in the health sciences in general.(5-7) Furthermore, it permits reinforcing systematic quantitative research that use said statistical tests, thus, providing greater inferential property to meta-analytical studies, and becoming an important methodological contribution inclusively for future articles in this journal.
